# Factors associated with self-rated health among mineworkers in Zambia: a cross-sectional study

**DOI:** 10.1186/s41182-021-00300-8

**Published:** 2021-02-01

**Authors:** Mayumi Ohnishi, Backsion Tembo, Rieko Nakao, Emi Matsuura, Wakako Fujita

**Affiliations:** 1 Nagasaki University Graduate School of Biomedical Sciences, 1-7-1 Sakamoto, Nagasaki, 852-8520 Japan; 2 Department of Chemistry, School of Natural Sciences, University of Zambia, Lusaka, Zambia

**Keywords:** Mineworkers, Self-rated health, Safety, Human security, Zambia

## Abstract

**Background:**

This study aims to examine miners’ working conditions and self-rated health status in copper mines in Zambia and to identify the conditions and factors necessary to improve the safety and health of mineworkers.

**Methods:**

A cross-sectional study using a self-administered questionnaire was conducted anonymously among copper mineworkers in Zambia in 2015 and 2016. Five targeted mining companies among 33 were introduced by the Mineworkers’ Union of Zambia. Study participants were recruited at the waiting space for underground work, waiting rooms of company clinics/hospitals, and/or at training sessions, which were places permitted by the target companies to perform data collection via convenience sampling. Bivariate analyses (e.g., *t* tests, Kruskal-Wallis tests, chi-square tests, or Cochran-Armitage tests) and logistic regression analysis were used to analyze differences in demographic characteristics and to compare their working conditions, health conditions, safety management at the workplace, and training opportunities by employment status.

**Results:**

In total, 338 responses were analyzed. Regular employees had better working conditions, including higher incomes (*P* = 0.001), more likely to be guaranteed sickness insurance by the company (*P* < 0.001), paid holidays (*P* = 0.094), and sick leave (*P* = 0.064), although the difference was not statistically significant. Mineworkers’ decreased self-rated health was determined by job category (adjusted odds ratio [AOR], 0.41; 95% confidence interval [CI], 0.21, 0.82; *P* = 0.012). Having experienced violence from the boss/manager (AOR, 0.54; 95% CI, 0.32, 0.91; *P* = 0.020) was negatively associated with better self-rated health in the crude odds ratio.

**Conclusions:**

Among mineworkers in Zambia, nonunderground work and not having experienced violence from their boss/manager contributed to increased self-rated health. From the perspective of psychological safety and human security, the management of safety and the working environment, including human resource management and preventing harassment/violence, should be assured, especially for underground mineworkers.

## Background

Previous studies revealed significant associations between underground mine work-related musculoskeletal injuries and ergonomic risk factors, such as working with a bent back and grasping objects [1, 2]. The fatality rate is high in the mining industry, particularly at underground sites. The most common cause of fatal injuries is falling rock in underground mines. The most frequent mechanism of injury is the handling of tools and materials, and the most commonly injured body parts are the hands and fingers in Zambia [3]. A study from the USA reported that slips and falls, electric injuries, the use of mining equipment, working in underground mining, worker age, and occupational experience were predictors of lost-time injuries in the mining industry, but there is insufficient evidence regarding mental health hazards [4].

Worldwide, working as a mineworker increases the risk of not only silicosis due to exposure to silica dust but also pulmonary tuberculosis regardless of the type of mine, such as coal, copper, gold, or others [5–7]. A study from South Africa demonstrated that there was a higher risk of mortality in the year after leaving mine work than in the general population [8]. Even in the USA, the prevalence of pneumoconiosis among coal mineworkers has been increasing [9], although mineworkers who have high compliance with health regulations are less likely to report lung disease [10]. Mineworkers’ safety and health challenges also include hearing loss/problems [2, 11, 12], sleep deprivation [13], and heart strain [14].

Previous studies have recommended safety- and health-promotion programs, including injury prevention and wellness programs, in the mining industry due to the high prevalence of injuries and mortality and adverse outcomes due to lost time [15, 16], although the implementation and feasibility of these programs have not been sufficiently guaranteed. Furthermore, an international shift toward using contract labor and extended workdays produces risks of long working hour injuries [17].

In recent decades, the safety and health of workers have received inadequate attention in Sub-Saharan African countries, including Zambia, due to the primary focus on infectious disease control, including HIV/AIDS, maternal and child health, and reproductive health [18]. However, the conditions surrounding workers have become increasingly complex and multifaceted in the face of increasing investment from foreign private sectors, while the implementation of policies and regulations to ensure safe and healthy working environments have lagged behind in low- and middle-income countries, including those of Sub-Saharan Africa. Up to several thousands of workers may work in a single copper mine in Zambia; for example, the largest four copper companies alone employed 56,300 individuals in 2012, including this study’s location [19]. The country, situated in the south of the African continent, is rich in mineral resources, and copper mining is the lifeblood of the Zambian economy. The rate of Zambia’s economic growth was 3.8% in 2018 [20]. According to a report by the International Labor Organization (ILO) in 2013, 26% of the 6000 cases of work-related illnesses and injuries reported between 2003 and 2007 took place in the mining industry. Increasing reports of health hazards among workers resulting from poor working environment violations of human rights are also being presented by international human rights organizations [21]. Furthermore, 12.4% of the reproductive population in Zambia is estimated to be HIV positive [22], which, together with tuberculosis, can potentially have a devastating impact on the country’s labor force. Although miners in Zambia enjoy one of the more stable working environments among similar Sub-Saharan African countries, their health and welfare needs remain uninvestigated. Studies on their health have been limited, with little attention given to risk prevention, health promotion, and health literacy. In addition, we have limited information regarding training opportunities in safe and healthy work environments and supervision, including human relationships such as workplace harassment and/or violence, among mineworkers who work under stressful conditions.

This study focuses on workers in the mining industry, which has enjoyed relatively stable industrial relations via large-scale labor unions. The study examines their working conditions and health status and identifies the conditions and factors necessary to strengthen the individual- and organizational-level safety and health of mineworkers.

## Methods

A cross-sectional study was conducted using a self-administered questionnaire distributed to mineworkers in the Copperbelt and Northwestern Provinces, Zambia, and data collection was performed in 2105 and 2016, respectively. The questionnaire included information on demographic characteristics in addition to working conditions, health and health check-up conditions, safety management and supervision at the workplace, and training opportunities. In addition, there was a free description space in the questionnaire, and the mineworkers could mention anything related to work. The questionnaire was prepared by the authors through interviews with mineworkers before the initiation of this study; the pretrial interviews were conducted at a company not included in the target companies in this study.

Five target mining companies among 33 companies were introduced by the Mineworkers’ Union of Zambia, and the researchers obtained permission to perform this study from the target mining companies in advance. The researchers recruited study participants at the waiting space for underground work, waiting rooms of company clinics/hospitals, and/or at the time of training sessions, which were places permitted to perform data collection by the target companies via convenience sampling in 2015 and 2016. The study participants recruited from the waiting rooms of company clinics/hospitals were mineworkers who visited the clinics/hospitals for their health check-ups. The study was conducted anonymously (both the study participants’ names and the companies’ names). The study participants completed the questionnaire after receiving oral and written explanations of the study objectives, procedures, data collection and management, publication, confidentiality, and ethical considerations regarding participation or refusal to participate in the study. Depositing the completed questionnaire in the collection box was deemed to represent consent to participate in the study. Before conducting the study, a research assistant or one of the authors gave an oral and written explanation of the study to and obtained written consent from the president and/or responsible human resources management of the study participants’ companies. No incentives were provided for the study participants.

Bivariate analyses, such as *t* tests, Kruskal-Wallis tests, chi-square tests or Cochran-Armitage tests, and logistic regression analysis were used to analyze the differences in the demographic characteristics and to compare their working conditions, health conditions, safety management at the workplace, and training opportunities by employment status using IBM SPSS (ver. 22). The significance level was set at 5%. In addition, a free listing was used to demonstrate free descriptions regarding working conditions and environment.

The study was approved by the University of Zambia Biomedical Research Ethics Committee (authorization number: 002-10-15) and the Ethical Committees of Nagasaki University Graduate School of Biomedical Sciences (authorization number: 15042404).

## Results

Among the mineworkers who were asked to participate in the study, none refused to complete the questionnaire after learning about the study procedures and ethical considerations. Finally, a total of 383 mineworkers submitted the questionnaire in the collection box. Twenty-five female respondents and respondents without sex information were excluded from the analysis because most female respondents were engaged in administrative work and/or ground staff. Twenty respondents lacked information regarding age, educational status, and employment status and were also excluded from the analysis. Therefore, all respondents that were included in the analysis were Zambian males. Table 1 demonstrates a difference in the respondents’ demographic characteristics by employment status, such as regular employment (*n* = 213) and contract employment (*n* = 125). Contract employees were younger than regular employees (*t* test, *P* = 0.001), more likely to be single or not living with a partner (chi-square test, *P* < 0.001), and more likely to drink alcoholic beverages (chi-square test, *P* = 0.043).

**Table 1:** Demographic characteristics and daily life habits of the study participants (*n* = 338)

	Regular employees (*n* = 213)		Contract employees (*n* = 125)		*P* value
	Mean (SD)		Mean (SD)		
	*n*	%	*n*	%	
Age (years)	39.4 (8.1)		36.1 (9.0)		0.001^a^
Educational status					
Did not complete high school	22	10.5	20	16.0	0.523^b^
Completed high school	102	48.6	53	42.4	
Completed college/university	86	41.0	52	41.6	
Religion					
Roman Catholic	55	26.6	35	28.5	0.280
Protestant/other Christian	138	66.7	74	60.2	
Muslim/others	14	6.8	14	11.4	
Marital status					
Single/not living with a partner	18	8.6	45	36.6	< 0.001
Married/living with a partner	192	91.4	79	63.7	
Smoking					
No	199	93.9	112	90.3	0.232
Yes	13	6.1	12	9.7	
Alcohol consumption					
No	115	55.0	54	43.5	0.043
Yes	94	45.0	70	56.5	
Playing sports as leisure/exercise					
No	34	16.1	26	20.8	0.278
Yes	177	83.9	99	79.2	

A *t* test^a^, chi-square test, or Cochran-Armitage test^b^ was conducted

Missing values were excluded from the analysis

Table 2 shows the health conditions and health- and safety-related conditions at the workplace. Regular employees were more likely to be committed to underground work than contract employees (chi-square test, *P* < 0.001). The former were also more likely to be guaranteed sickness insurance by the company (chi-square test, *P* < 0.001), paid holidays (chi-square test, *P* = 0.094), and sick leave (chi-square test, *P* = 0.064), although the difference was not statistically significant. The income in Kwacha of regular workers (mean ± standard deviation, 4681 ± 1994) was higher than that of contract workers (3595 ± 3197) (*t* test, *P* < 0.001). There was a statistically significant difference in income by educational status (did not complete high school: 3197 ± 1422, completed high school: 4328 ± 2250, and completed college/university: 4571 ± 2962, respectively, Kruskal-Wallis test, *P* = 0.002). Among respondents who worked the night shift, there was no statistically significant difference in hours of night shift work (mean ± standard deviation) between regular (10.7 ± 6.8) and contract (11.0 ± 5.3) employees (*t* test, *P* = 0.763). Among respondents who worked overtime, there was also no statistically significant difference in hours of overtime between regular (13.1 ± 13.0) and contract (12.4 ± 11.4) employees (*t* test, *P* = 0.730).

**Table 2:** Working conditions of the study participants (*n* = 338)

	Regular employees (*n* = 213)		Contract employees (*n* = 125)		*P* value
	Mean (SD)		Mean (SD)		
	*n*	%	*n*	%	
Job category					
Nonunderground work	102	47.9	87	69.6	< 0.001
Underground work	111	52.1	38	30.4	
Years of working	13.9 (8.0)		10.0 (8.0)		< 0.001 ^a^
Income per month (Kwacha)	4681 (1994)		3595 (3197)		< 0.001 ^a^
Working hours per week					
40 h or more	176	85.4	103	85.1	0.938
Less than 40 h	30	14.6	18	14.9	
Nightshift work					
No	108	50.9	62	50.8	0.983
Yes	104	49.1	60	49.2	
Overtime work					
No	70	33.2	45	39.1	0.282
Yes	141	66.8	70	60.9	
Breaks during work					
No break	50	25.9	23	18.9	0.113
Every 1 to 3 h	56	29.0	31	25.4	
Once (30 min to 1 h) per shift	50	25.9	47	28.5	
Others	37	19.2	21	17.2	
Provided a meal/snack at workplace					
No	94	46.3	23	19.2	< 0.001
Yes	109	53.7	97	80.8	
Guaranteed paid holidays					
No paid holidays	31	17.6	27	25.7	0.094 ^b^
Less than 20 days	22	12.5	18	17.1	
20 days or more	123	69.9	60	57.1	
Took a paid holiday in the last year					
No	63	33.7	47	45.6	0.045
Yes	124	66.3	56	54.4	
Sickness insurance provided by the company					
No	43	21.4	60	52.2	< 0.001
Yes	158	78.6	55	47.8	
Guaranteed sick leave					
No	61	31.6	46	42.2	0.064
Yes	132	68.4	63	57.8	
Experience of taking sick leave					
No	114	60.6	64	58.7	0.744
Yes	74	39.4	45	41.3	

A *t* test^a^, chi-square test, or Cochran-Armitage test^b^ was conducted

Missing values were excluded from the analysis

^c^1 US$ = approximately 9.8 Zambian Kwacha at the time of data collection

Table 3 demonstrates self-rated health and health- and safety-related conditions at the workplace by employment status. A total of 330 study participants provided responses of self-rated health, and 241 (73.0%) of them reported “very good” or “good” self-reported health. There was no significant difference in the self-rated health conditions between regular employees and contract employees (chi-square test, *P* = 0.625). Regular employees were more likely to have availability of health check-ups by the company in the last year than contract employees (chi-square test, *P* = 0.078), although the difference was not statistically significant. However, other conditions, such as experiencing accidents and/or violence at the workplace and supervision, did not show statistically significant differences.

**Table 3:** Health conditions and health- and safety-related conditions in the workplace (*n* = 330)

	Regular employees (*n* = 213)		Contract employees (*n* = 125)		*P* value
	*n*	%	*n*	%	
Self-rated health					
Average/bad	58	27.9	31	25.4	0.625
Very good/good	150	72.1	91	74.6	
Availability of health check-ups by the company					
No	24	11.8	22	18.2	0.113
Yes	179	88.2	99	81.8	
Received a health check-up in the last year					
No	25	12.0	13	10.7	0.078
Yes, by the company	168	80.4	90	73.8	
Yes, by self-provision	16	7.7	19	15.6	
Occurrence of a health problem/concern					
No	130	66.7	74	63.2	0.539
Yes	65	33.3	43	36.8	
Experienced an accident in the workplace					
No	129	65.5	77	65.8	0.953
Yes	68	34.5	40	34.2	
Experienced violence from the boss					
No	99	50.8	53	46.9	0.513
Yes	96	49.2	60	53.1	
Experienced violence from colleagues					
No	142	72.8	78	69.6	0.552
Yes	53	27.2	34	30.4	
Supervision in the workplace					
No	41	19.7	16	13.3	0.142
Yes	167	80.3	104	86.7	
Self-evaluation of safety of the workplace					
Not safe	63	31.0	30	25.0	0.247
Safe	140	69.0	90	75.0	

Chi-square tests were conducted

Missing values were excluded from the analysis

Training opportunities regarding working conditions when the employees began working were more likely to be guaranteed for regular employees than contract employees, although the proportions were not high (37.1% and 19.6%, respectively), and other training opportunities at the beginning of work were provided to approximately 20% of both regular and contract employees. Only training about safety in the workplace was provided to more than half of the workers at the beginning of work among both regular and contract employees. More than 90% of the respondents, both regular and contract employees, received training about safety management at the workplace after starting work (Table 4).

**Table 4:** Training opportunities (*n* = 338)

	Regular employees (*n* = 213)		Contract employees (*n* = 125)		*P* value
	*n*	%	*n*	%	
Training when the employees began work					
Working conditions					
No	124	62.9	90	80.4	0.001
Yes	73	37.1	22	19.6	
Working skills					
No	150	76.1	95	84.8	0.070
Yes	47	23.9	17	15.2	
How to use equipment for work					
No	156	79.2	89	79.5	0.954
Yes	41	20.8	23	20.5	
Safety in the workplace					
No	98	49.7	41	36.6	0.026
Yes	99	50.3	71	63.4	
Health					
No	153	77.7	96	85.7	0.086
Yes	44	22.3	16	14.3	
Training about safety management in the workplace after starting work					
No	10	5.0	6	5.1	0.952
Yes	191	95.0	111	94.9	

Chi-square tests were conducted

Missing values were excluded from the analysis

Table 5 shows the factors associated with self-rated health that were calculated using self-rated health as the dependent variable and age, income, educational status, employment status, job category, provision of meal/snack at the workplace, paid holiday, sickness insurance, experience of violence, and supervision in the workplace as independent variables using logistic regression analysis. Regardless of employment status, working underground (adjusted odds ratio [AOR], 0.41; 95% confidence interval [CI], 0.21, 0.82; *P* = 0.012) was negatively associated with better self-rated health in the logistic regression analysis. Although significant associations were not found after adjustment, higher educational status, being provided with a meal/snack at the workplace, and receiving supervision contributed to better self-rated health. On the other hand, experiencing violence from the boss/manager was negatively associated with better self-rated health in the crude odds ratio (OR, 0.54; 95% CI, 0.32, 0.91; *P* = 0.020).

**Table 5:** Factors associated with self-rated health among mineworkers

	OR	95% CI	*P* value	AOR	95% CI	*P* value
Age (continuous valuable)	0.98	0.95, 1.01	0.121	0.99	0.95, 1.03	0.691
Income per month (Kwacha)	1.01	0.57, 1.79	0.966	1.00	1.00, 1.00	0.977
Educational status						
Did not complete high school	1			1		
Completed high school	2.64	1.27, 5.48	0.009	3.09	1.04, 9.17	0.041
Completed college/university	2.81	1.34, 5.92	0.006	3.11	1.02, 9.47	0.109
Employment status						
Regular employees	1			1		
Contract employees	1.14	0.68, 1.89	0.625	0.98	0.43, 2.21	0.961
Job category						
Nonunderground work	1			1		
Underground work	0.41	0.25, 0.68	< 0.001	0.41	0.21, 0.82	0.012
Provided a meal/snack at the workplace						
No	1			1		
Yes	2.13	1.29, 3.51	0.003	1.85	0.92, 3.73	0.084
Guaranteed paid holidays						
No paid holidays	1			1		
Less than 20 days	1.07	0.42, 2.71	0.884	0.89	0.27, 2.88	0.842
20 days or more	0.98	0.50, 1.92	0.945	0.69	0.29, 1.63	0.395
Sickness insurance provided by the company						
No	1			1		
Yes	1.66	0.98, 2.79	0.058	1.65	0.75, 3.66	0.216
Experienced violence from the boss						
No	1			1		
Yes	0.54	0.32, 0.91	0.020	0.62	0.32, 1.23	0.172
Supervision in the workplace						
No	1			1		
Yes	2.85	1.57, 5.17	0.001	1.96	0.86, 4.48	0.111

A logistic regression analysis was conducted

Missing values were excluded from the analysis

Table 6 demonstrates a free description regarding the working conditions and environment. The free description spaces included 8 statements related to supervision and/or inspection, such as “Employees work properly without pressure. Sometimes accidents come from pressure from bosses or supervisors, so don’t put too much pressure on employees (by a contract worker)” and “Some supervisors think that the worker is just bluffing, doesn’t want to work while he is sick. As a result, other workers fear disclosing how they are feeling, and at the end of the day, the workers collapsed (by a contract worker).” There were also 23 statements regarding training, such as “Working in the mining industry is crucial. The company must form a team to train employees to avoid being involved in accidents (by a contract worker).” Regarding working conditions, including safety in the workplace, such as dust, heat, and noise (96 statements), salary/payment (79 statements), and holidays/leave (7 statements), respondents provided comments such as “The working conditions are very poor, and we are not allowed to take annual leave; instead, we are paid, so we do not have enough rest. The company has no salary structure; hence, people are not paid according to their qualifications (by a contract worker)” and “Most employees find themselves in unsafe and unhealthy situations due to pressure of work from supervisors combined with personal, social and economic challenges (by a contract worker).”

**Table 6:** Free description regarding working conditions and environment

	Regular employees (*n* = 213)	Contract employees (*n* = 125)
Insufficient salary/payment	45	34
Need to improve safety environment	40	20
Too much dust	21	7
Need more training	18	4
Need appropriate personal protective equipment (PPE)	15	7
Need to improve healthcare of workers	12	13
Need more motivation/incentive by the company/employer	8	6
Need to improve working condition/environment in general	5	6
Insufficient meal/snack during working hours	4	2
Need appropriate supervision/inspection	4	4
Too much pressure/intimidation/forced task	4	3
Too much hot	3	2
Need a route for escape/evacuation	3	1
Too much noisy	2	1
Need to revise/improve regulation on the mine	2	0
No freedom	2	0
Inappropriate overtime (without remuneration, no rest, etc.)	2	3
Improve general sanitation and water sanitation	2	0
No day-off/leave/holiday	2	5
Challenges of relationships with colleagues	1	0
Challenges of relationships with boss	1	1
Need more welfare	1	0
No enough information on labor laws	1	0
High medical cost	1	0
Workers feel fear of disclose	0	1
Racism	0	1
Gender discrimination	0	1

## Discussion

According to the findings in this study, in the copper mines of Zambia, regular employees had better working conditions, including higher income, paid holidays, and sickness insurance, than contract employees. However, mineworkers’ self-rated health was determined by job category, such as working underground.

According to the Mineworkers’ Union of Zambia, among mineworkers in Zambia, regular employees are protected in regard to income and working conditions, including paid holidays, health check-ups, and sickness insurance. These conditions should be fundamentally guaranteed for decent work among mineworkers regardless of employment status. However, both regular and contract workers mentioned inappropriateness of working conditions such as insufficient salary, unsafe environment at work place, and lack of adequate supervision, training, and equipment for safety control, in the free descriptions regardless of employment status. In this study, mineworkers could not honestly disclose their health conditions and illness because they were afraid that their boss/manager would regard them as false illnesses and/or loafing. In addition, the mineworkers could not assert opinions regarding safety due to fear of receiving criticisms and/or intimidation from their boss/manager. This kind of psychological pressure and stress from the boss/manager may produce unsafe and/or unhealthy working conditions. Especially for underground mineworkers, safety in the work environment should be assured, not only by enhancing physical conditions, including temperature and noise but also by managing human resources and preventing harassment/violence from the perspective of psychological safety and human security. It is important to consider a participatory approach whereby both workers and employees engage in policymaking and risk prevention. Regarding psychological safety and human security, in addition to guaranteeing these conditions, safety and working environment management, including human resource management and the prevention of harassment/violence, should be assured, especially for underground mineworkers.

A previous study reported that higher income, low stress, and higher job satisfaction were factors associated with early return to work after injuries among mineworkers [23]. From the perspective of the effort-reward imbalance model, higher income is an essential factor that is associated with better quality of life among mineworkers [24]. In this study, higher income was associated with regular employment and higher educational status, but self-rated health was not associated with employment status, such as regular or contract employment. In general, higher income is associated with higher educational status [25], but there were complex relationships among employment status, job categories, working conditions, and educational status in this study. For example, underground mineworkers committed to heavy workloads and nightshift work with uncomfortable conditions, including high temperatures and noisy settings, regardless of educational status. Additionally, having experienced violence that might have resulted in stressful working conditions was associated with self-rated health. Individuals who reported lower self-rated health may have received higher income if their psychological safety was not protected. Regarding health personnel management, the inadequate number and distribution of personnel and mental fatigue in health personnel in low- and middle-income countries are serious challenges [26–28]. Improving the motivation of health personnel is essential for retention, but financial incentives alone are not sufficient to motivate them. The appropriate distribution of human resources, necessary equipment, and financing are indispensable, and functioning management and the maintenance of infrastructure are also required [29]. A study from Senegal reported that the provision of a permanent contract was the most important factor for retaining employees in rural and challenging regions, following the availability of necessary equipment in working facilities and the provision of training opportunities [30]. In the mining industries, the situation may be similar. The satisfaction of temporal financial needs as well as comfortable working environments/conditions, including a balanced and proper salary and guaranteed sustainability of these working conditions, should be fundamental for both individual workers and organizations. In this study, training opportunities did not differ between regular and contract employees and did not contribute to self-rated health, although previous studies have indicated that training opportunities were one of the key factors for retaining employees [31]. Additionally, training opportunities, especially entry training at the start of employment at mining companies, were very limited in this study. This may be due to the conditions of the economic market, different levels of professionalism, and background educational status. Further studies should be conducted to determine reasonable factors and explanations.

According to the concept of decent work by the ILO [32, 33], psychological health in the workplace and work conditions should not be neglected, not only for safety and health of workers but also to maintain equity and social justice. Holistic approaches, such as achieving decent working conditions and appropriate supervision, could guarantee comprehensive well-being, including safety and health [34], and could strengthen individual and organizational potentiality in underserved settings, such as Zambian mine workplaces. Although there are stressful conditions, including human relationships and workplace harassment/violence, functioning supervision can mitigate mineworkers’ complaints. Therefore, training for both mineworkers and supervisors should be required to produce decent working conditions. For that reason, it is important that a participatory approach be considered, whereby both workers and employees engage in policymaking and risk prevention.

This study has several limitations. First, biological measures were not evaluated to assess objective health status. Self-rated health was used to evaluate mineworkers’ health status. However, a previous study demonstrated that self-rated health could be a measurement of health status [35]. Second, this study did not assess the causal factors related to mineworkers’ health status. Third, this study did not fully discuss the contribution of mental health conditions and stress coping to self-rated health. Fourth, details regarding working hours and shifts were not evaluated in this study. According to interviews with administrative officers of the mining companies conducted by the authors, underground workers continue a rotation of nightshift work for 10 days, followed by a 2- or 3-day holiday, and then dayshift work for 10 days, at one of the target companies in this study. However, each company had its own regulations, and the work shift varied depending on the type of work. Further studies are required to describe the relationships between working conditions/environments and mineworkers’ safety and health status. Fifth, the questionnaire survey was limited in terms of evaluating the details of “violence” in the workplace. Some respondents considered only physical violence as “violence,” and others might have included verbal and/or psychological violence and other types of harassment. Sixth, the results were not fully obtained from a representative population of mineworkers in Zambia because there were difficulties in accessing the target population.

## Conclusions

The findings from this study demonstrated that accidents and harassment/violence committed by a boss/manager in the workplace were common in mining companies. Among mineworkers in Zambia, nonunderground work and not having experienced violence from the boss/manager contributed to increased self-rated health. From the perspective of psychological safety and human security, management of safety and the working environment, including human resource management and the prevention of harassment/violence, should be assured, especially for underground mineworkers. Appropriate responses to workplace harassment/violence and supervision can contribute to providing balanced working conditions among mineworkers in stressful environments.

## Data Availability

The datasets used and/or analyzed during the current study are available from the corresponding author on reasonable request.

## Abbreviations

AIDS: Acquired immunodeficiency syndrome
AOR: Adjusted odds ratio
CI: Confidence interval
HIV: Human immunodeficiency virus
ILO: International Labor Organization
